# Enhanced Orai1 and STIM1 expression as well as store operated Ca^2+^ entry in therapy resistant ovary carcinoma cells

**DOI:** 10.18632/oncotarget.2035

**Published:** 2014-05-28

**Authors:** Sebastian Schmidt, Guoxing Liu, Guilai Liu, Wenting Yang, Sabina Honisch, Stavros Pantelakos, Christos Stournaras, Arnd Hönig, Florian Lang

**Affiliations:** ^1^ Department of Physiology, University of Tübingen, D72076 Tübingen, Germany; ^2^ University of Crete Medical School, Department of Biochemistry, Voutes, Heraklion, 71110, Greece; ^3^ Department of Gynecology, University Hospital of Würzburg, D97080 Würzburg, Germany

**Keywords:** Ca2+ release activated Ca2+ channel, SOCE, Akt, SH-6, 2-APB, apoptosis

## Abstract

Mechanisms underlying therapy resistance of tumor cells include protein kinase Akt. Putative Akt targets include store-operated Ca^2+^-entry (SOCE) accomplished by pore forming ion channel unit Orai1 and its regulator STIM1. We explored whether therapy resistant (A2780cis) differ from therapy sensitive (A2780) ovary carcinoma cells in Akt, Orai1, and STIM1 expression, Ca^2+^-signaling and cell survival following cisplatin (100μM) treatment. Transcript levels were quantified with RT-PCR, protein abundance with Western blotting, cytosolic Ca^2+^-activity ([Ca^2+^]i) with Fura-2-fluorescence, SOCE from increase of [Ca^2+^]i following Ca^2+^-readdition after Ca^2+^-store depletion, and apoptosis utilizing flow cytometry. Transcript levels of Orai1 and STIM1, protein expression of Orai1, STIM1, and phosphorylated Akt, as well as SOCE were significantly higher in A2780cis than A2780 cells. SOCE was decreased by Akt inhibitor III (SH-6, 10μM) in A2780cis but not A2780 cells and decreased in both cell lines by Orai1 inhibitor 2-aminoethoxydiphenyl borate (2-ABP, 50μM). Phosphatidylserine exposure and late apoptosis following cisplatin treatment were significantly lower in A2780cis than A2780 cells, a difference virtually abolished by SH-6 or 2-ABP. In conclusion, Orai1/STIM1 expression and function are increased in therapy resistant ovary carcinoma cells, a property at least in part due to enhanced Akt activity and contributing to therapy resistance in those cells.

## INTRODUCTION

Cytosolic Ca^2+^ activity participates in the regulation of a variety of fundamental cellular mechanisms including excitation, exocytosis, migration, cell proliferation and cell death [1-5]. Mechanisms contributing to the regulation of cytosolic Ca^2+^ concentration include Ca^2+^ release from intracellular stores and subsequent activation of store operated Ca^2+^ entry (SOCE) or Ca^2+^ release activated Ca^2+^ channel I_CRAC_, which are accomplished by the pore forming Ca^2+^ channel subunits Orai1, Orai2 and/or Orai3 [6-10] as well as their regulators STIM1 and/or STIM2 [11-15]. Orai1 and STIM1 are expressed in tumor cells and may well contribute to the survival of therapy resistant cells [16-20].

STIM1 and Orai1 upregulate SOCE via activation of Akt/mTOR in human pulmonary arterial smooth muscle cells [21]. In addition Orai1 and thus SOCE is up-regulated in both mast cells [22] and platelets [23] by the serum & glucocorticoid inducible kinase SGK1, which counteracts Orai1 degradation by the ubiquitin ligase Nedd4-2 [22] and stimulates NF-κB dependent Orai1 transcription [24]. SGK1 targets are in large part shared by PKB/Akt isoforms [25]. Thus, it is feasible that Orai1 is similarly regulated by PKB/Akt isoforms.

The present study explored whether Orai1 and STIM1 are expressed in ovary carcinoma cells and whether their expression and function differs between therapy resistant and therapy sensitive ovary carcinoma cells. To this end, Orai1 and STIM1 transcript and protein expression, SOCE as well as apoptosis following cisplatin treatment were determined in therapy resistant and therapy sensitive ovary carcinoma cells.

## RESULTS

**Fig. 1:**
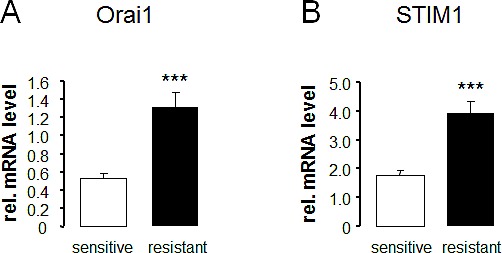
Transcript levels of Orai1 and STIM1 in therapy sensitive and therapy resistant ovary carcinoma cells Arithmetic means ± SEM (n = 9) of Orai1 (A) and STIM1 (B) transcript levels in therapy sensitive (white bars) and therapy resistant (black bars) ovary carcinoma cells. *** (p<0.001) indicates statistically significant difference from therapy sensitive ovary carcinoma cells (ANOVA).

RT-PCR was employed to explore whether ovary carcinoma cells transcribe Orai1 and/or STIM1 and whether the transcript levels are different between therapy sensitive A2780 and therapy resistant A2780cis ovary carcinoma cells. As illustrated in Fig.1, both cell lines express Orai1 and STIM1. The transcript levels were, however, significantly higher in therapy resistant A2780cis than in therapy sensitive A2780 ovary carcinoma cells.

**Fig. 2:**
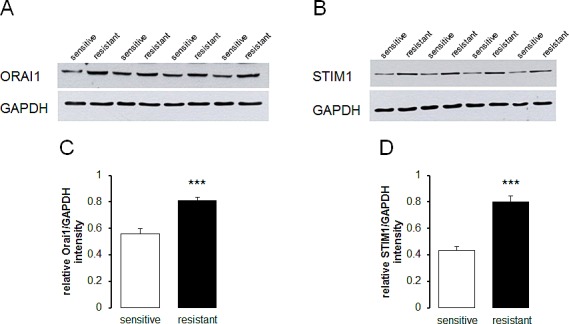
Protein abundance of Orai1 and STIM1 in therapy sensitive and therapy resistant ovary carcinoma cells A,B. Original Western blot of whole tissue lysate protein of Orai1 (A) and STIM1 (B) as well as GAPDH in therapy sensitive and therapy resistant ovary carcinoma cells. C,D. Arithmetic means ± SEM (n = 4) of the Orai1/GAPDH (C) and STIM1/GAPDH (D) protein abundance ratios in therapy sensitive (white bars) and therapy resistant (black bars) ovary carcinoma cells. *** (p<0.001) indicates statistically significant difference from therapy sensitive ovary carcinoma cells (ANOVA).

In line with the differences in Orai1 and STIM1 transcript levels, Western blotting analysis revealed similar differences in protein abundance. Indeed, as illustrated in Fig. 2, the Orai1 and STIM1 protein expression was significantly higher in therapy resistant A2780cis than in therapy sensitive A2780 ovary carcinoma cells.

**Fig. 3:**
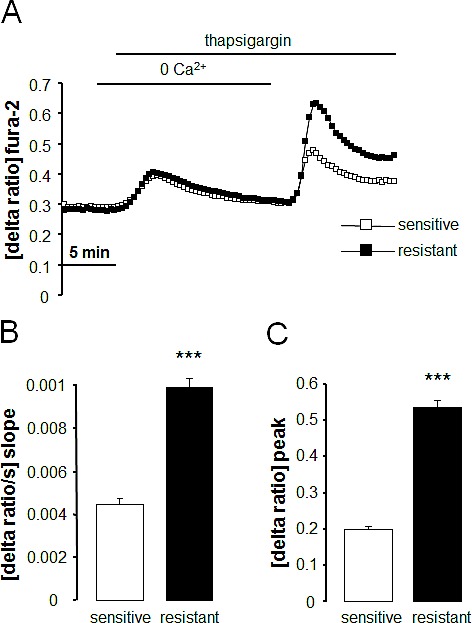
Intracellular Ca^2+^ release and store operated Ca^2+^ entry (SOCE) in therapy sensitive and therapy resistant ovary carcinoma cells A. Representative tracings of fura-2-fluorescence-ratio in fluorescence spectrometry before, during and after Ca^2+^ depletion with subsequent addition of thapsigargin (1 μM) in therapy sensitive (white squares) and therapy resistant (black squares) ovary carcinoma cells. B,C. Arithmetic means (± SEM, n = 7, each experiment 10-30 cells) of slope (B) and peak (C) increase of fura-2-fluorescence-ratio following readdition of extracellular Ca^2+^ in therapy sensitive (white bars) and therapy resistant (black bars) ovary carcinoma cells. *** (p<0.001) indicates statistically significant difference from therapy sensitive ovary carcinoma cells (ANOVA).

**Fig. 4:**
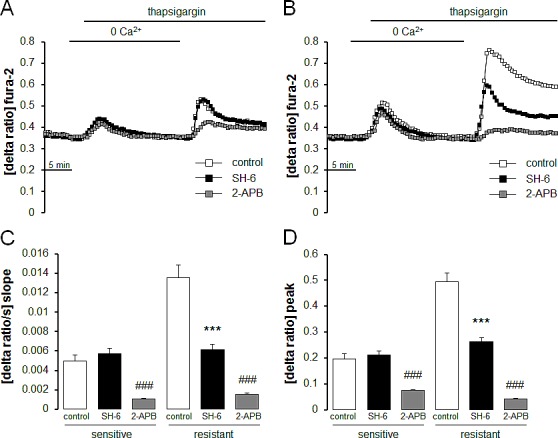
Effect of Akt inhibitor SH-6 and Orai1 inhibitor 2-APB on intracellular Ca^2+^ release and store operated Ca^2+^ entry (SOCE) in therapy sensitive and therapy resistant ovary carcinoma cells A,B. Representative tracings of fura-2- fluorescence-ratio in fluorescence spectrometry during and after Ca^2+^ depletion with subsequent thapsigargin (1 μM) addition in therapy sensitive (A) and therapy resistant (B) ovary carcinoma cells without (white squares) and with presence of Akt inhibitor SH-6 (10 μM, black squares) or Orai1 inhibitor 2-APB (50μM, gray squares). C,D. Arithmetic means (± SEM, n = 5-6, each experiment 10-30 cells) of slope (C) and peak (D) increase of fura-2-fluorescence-ratio following Ca^2+^ readdition in therapy sensitive (left bars) and therapy resistant (right bars) ovary carcinoma cells in the absence (white bars) and presence of Akt inhibitor SH-6 (10 μM, black bars) or Orai1 inhibitor 2-APB (50μM, gray bars). *** and ^###^(p<0.001) indicate statistically significant difference from absence of inhibitors(ANOVA).

Fura-2-fluorescence was employed in order to test whether the differences in Orai1 and STIM1 transcript levels and protein abundance were paralleled by corresponding differences in store operated Ca^2+^ entry. The Fura-2-fluorescence ratio prior to extracellular Ca^2+^ removal was similar in therapy sensitive A2780 (0.298 ± 0.003 a.u., n = 7) and therapy resistant A2780cis (0.309 ± 0.003 a.u., n = 7) ovary carcinoma cells. Addition of the store-depleting sarco-/endoplasmic reticulum Ca^2+^-ATPase (SERCA) inhibitor thapsigargin (1 μM) triggered release of Ca^2+^ from intracellular stores, leading to rapid, transient increase in cytosolic Ca^2+^ activity. The increase of intracellular Ca^2+^ concentration following thapsigargin treatment was again similar in therapy sensitive A2780 and therapy resistant A2780cis ovary carcinoma cells (Fig. 3). As illustrated in Fig. 3, the subsequent addition of extracellular Ca^2+^ was followed by a rapid increase of Fura-2-fluorescence in both cell types reflecting store operated Ca^2+^ entry (SOCE). Both, peak and slope of SOCE were significantly higher in therapy resistant A2780cis than in therapy sensitive A2780 ovary carcinoma cells. The Orai1 inhibitor 2-APB (50 μM) decreased the peak Ca^2+^ increase from 0.19 ± 0.02 arbitrary units (n = 6) to 0.07 ± 0.01 arbitrary units (n = 5) in A2780 ovary carcinoma cells and from 0.49 ± 0.03 arbitrary units (n = 6) to 0.04 ± 0.01 arbitrary units (n = 5) A2780cis ovary carcinoma cells (Fig. 4).

**Fig. 5:**
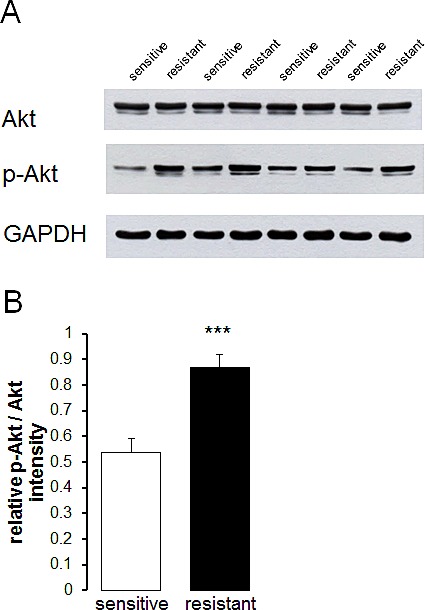
Protein abundance of Akt and p-Akt in therapy sensitive and therapy resistant ovary carcinoma cells A. Original Western blot of whole tissue lysate protein of total Akt and phosphorylated p-Akt as well as GAPDH in therapy sensitive and therapy resistant ovary carcinoma cells. B. Arithmetic means ± SEM (n = 4) of the p-Akt/Akt protein abundance ratios in therapy sensitive (white bars) and therapy resistant (black bars) ovary carcinoma cells. *** (p<0.001) indicates statistically significant difference from therapy sensitive ovary carcinoma cells (ANOVA).

Additional experiments attempted to elucidate mechanisms accounting for the differences in Orai1 and STIM1 abundance as well as SOCE between therapy resistant A2780cis and therapy sensitive A2780 ovary carcinoma cells. A candidate kinase was Akt. Thus, Western blotting analysis was employed to test whether Akt and p-Akt are differentially expressed in therapy resistant A2780cis and in therapy sensitive A2780 ovary carcinoma cells. As a result, the protein abundance of p-Akt/Akt was indeed significantly higher in therapy resistant A2780cis than in therapy sensitive A2780 ovary carcinoma cells (Fig. 5). To further analyze whether Akt activity was required for the differences in SOCE, Fura-2-fluorescence experiments were performed in the absence and presence of Akt inhibitor III (SH-6) (Fig. 4). Pretreatment with SH-6 (10 μM) did not significantly modify the Fura-2-fluorescence of therapy sensitive A2780 (0.35 ± 0.01 a.u., n = 6) and therapy resistant A2780cis (0.35 ± 0.01 a.u., n = 6) ovary carcinoma cells prior to triggering of SOCE. Moreover, the increase in cytosolic Ca^2+^ activity following addition of thapsigargin (1 μM) was in both cell types similar in the absence and presence of SH-6 (Fig. 4). However, the rapid increase of Fura-2-fluorescence following subsequent addition of extracellular Ca^2+^ was in therapy resistant A2780cis but not in therapy sensitive A2780 cells significantly blunted by SH-6 (Fig. 4). In the presence of SH-6 no statistically significant difference was observed in SOCE between therapy sensitive A2780 and therapy resistant A2780cis ovary carcinoma cells.

**Fig. 6:**
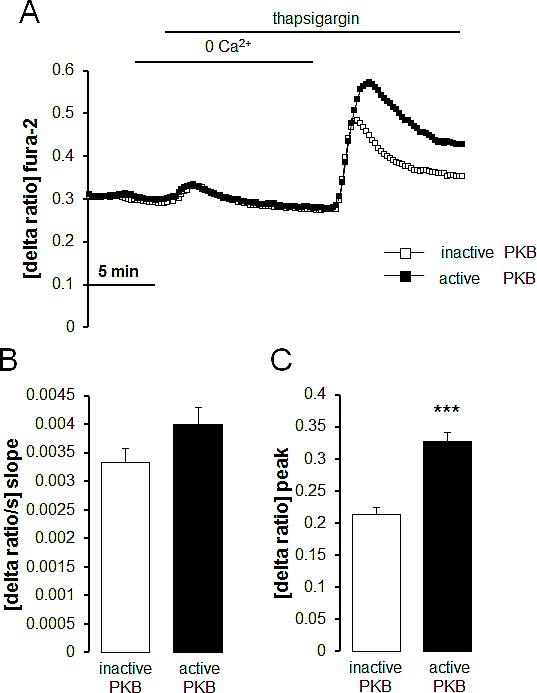
Intracellular Ca^2+^ release and store operated Ca^2+^ entry (SOCE) in therapy sensitive ovary carcinoma cells transfected with constitutively active Akt or inactive Akt A. Representative tracings of fura-2 fluorescence-ratio in fluorescence spectrometry during and after Ca^2+^ depletion with subsequent thapsigargin (1 μM) addition in therapy sensitive ovary carcinoma cells transfected with constitutively active (T308DS473D) Akt (black squares) or inactive (T308AS473A) Akt (white squares). B,C. Arithmetic means (± SEM, n = 5, each experiment 10-30 cells) of slope (B) and peak (C) increase of fura-2-fluorescence-ratio in therapy sensitive ovary carcinoma cells transfected with constitutively active (T308DS473D) Akt (black bars) or inactive (T308AS473A) Akt (white bars). *** (p<0.001) indicates statistically significant difference from therapy sensitive ovary carcinoma cells (ANOVA).

In order to further explore the influence of Akt on SOCE, intracellular Ca^2+^ release and SOCE were determined in therapy sensitive ovary carcinoma cells transfected with constitutively active Akt or inactive Akt. As illustrated in Fig. 6, SOCE was significantly higher following transfection with constitutively active (T308DS473D) Akt than following transfection with inactive (T308AS473A) Akt.

**Fig. 7:**
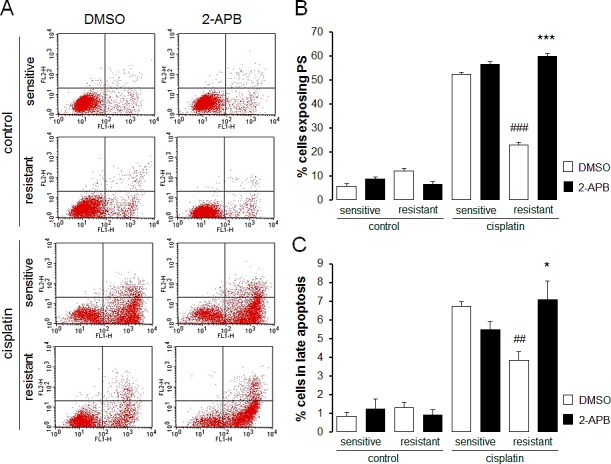
Effect of SOCE inhibitor 2-APB on cisplatin induced apoptosis of therapy sensitive and therapy resistant ovary carcinoma cells A. Original dot plots of a representative experiment of annexin V binding plotted against propidium iodide staining of therapy sensitive cells (sensitive) and therapy resistant ovary carcinoma cells (resistant) without (left panels) and with (right panels) a 24 h exposure to 50 μM 2-APB and with (cisplatin) and without (control) cisplatin (100 μM, 24 h) treatment. The cells without loss of membrane integrity and externalized phosphatidylserine at the cell surface appear on the lower left quadrant of the dot plot. B. Arithmetic means (± SEM, n =5-6) of the percentage of therapy sensitive (sensitive) and therapy resistant (resistant) ovary carcinoma cells binding Annexin V following 24 h exposure to DMSO (1‰, white bars) or 2-APB (50 μM, black bars) prior to (control) and following (cisplatin) treatment with cisplatin (100 μM, 24 h). *** (p<0.001) indicates statistically significant difference from respective value without 2-APB exposure, ^###^ (p<0.001) indicates statistical difference from therapy sensitive cells following exposure to DMSO (ANOVA). C. Arithmetic means (± SEM, n =5-6) of the percentage of therapy sensitive (sensitive) and therapy resistant (resistant) ovary carcinoma cells undergoing late apoptosis following 24 h exposure to DMSO (1‰, white bars) or 2-APB (50 μM, black bars) prior to (control) and following (cisplatin) treatment with cisplatin (100 μM, 24 h). * (p<0.05) indicates statistically significant difference from respective value without 2-APB exposure, ^#^ (p<0.05) indicates statistical difference from therapy sensitive cells following exposure to DMSO (ANOVA).

**Fig. 8:**
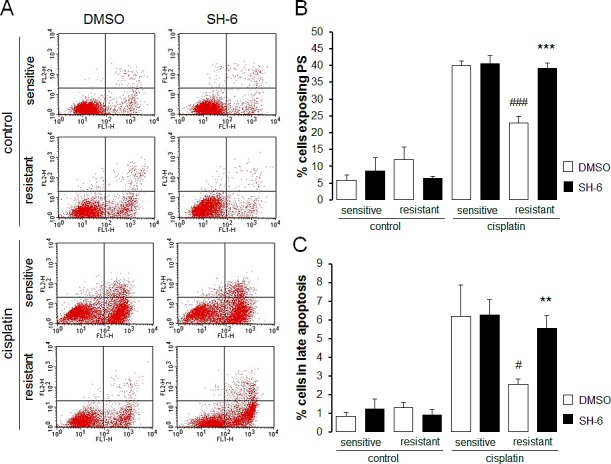
Effect of Akt inhibitor SH-6 on cisplatin induced apoptosis of therapy sensitive and therapy resistant ovary carcinoma cells A. Original dot plots of annexin V binding plotted against propidium iodide in therapy sensitive cells (sensitive) and therapy resistant (resistant) ovary carcinoma cells without (left panels) and with (right panels) a 24 h exposure to 10 μM SH-6 and without (control) and with (cisplatin) treatment with cisplatin (100 μM, 24 h). The cells without loss of membrane integrity and externalized phosphatidylserine at the cell surface appear on the lower left quadrant of the dot plot. B. Arithmetic means (± SEM, n = 6) of the percentage of therapy sensitive (sensitive) and therapy resistant (resistant) ovary carcinoma cells binding Annexin V following 24 h exposure to DMSO (1‰, white bars) or SH-6 (10 μM, black bars) prior to (control) and following treatment with cisplatin (100 μM, 24 h) (cisplatin). *** (p<0.001) indicates statistically significant difference from respective value without SH-6 exposure, ^###^ (p<0.001) indicates statistical difference from therapy sensitive cells following exposure to DMSO (ANOVA). C. Arithmetic means (± SEM, n = 6) of the percentage of therapy sensitive (sensitive) and therapy resistant (resistant) ovary carcinoma cells undergoing late apoptosis following 24 h exposure to DMSO (1‰, white bars) or SH-6 (10 μM, black bars) prior to (control) and following (cisplatin) treatment with cisplatin (100 μM, 24 h). ** (p<0.01) indicates statistically significant difference from respective value without SH-6 exposure, ^#^ (p<0.05) indicates statistical difference from therapy sensitive cells following exposure to DMSO (ANOVA).

In order to test whether Akt sensitive regulation of Orai1 influenced the sensitivity of the ovary carcinoma cells to therapy, the effect of cisplatin on apoptosis of therapy sensitive A2780 and therapy resistant A2780cis cells was tested in the absence and presence of either Akt inhibitor SH-6 or Orai1 inhibitor 2-aminoethoxydiphenyl borate (2-APB). As illustrated in Fig. 7 and Fig. 8, A2780cis cells were resistant to apoptosis induced by cisplatin when compared with sensitive cells. Both SH-6 (10 μM) and 2-ABP (50 μM) restored cisplatin sensitivity of resistant cells significantly to levels comparable with those observed in sensitive cells.

## DISCUSSION

The present study disclosed the expression of Orai1 and STIM1 in both, therapy sensitive A2780 and therapy resistant A2780cis ovary carcinoma cells. More importantly, the present observations revealed that expression of both, Orai1 and STIM1, was significantly higher in therapy resistant A2780cis than in therapy sensitive A2780 ovary carcinoma cells. The increased expression of Orai1 and STIM1 was paralleled by corresponding differences in store operated Ca^2+^ entry (SOCE) in those cells. The enhanced Orai1/STIM1 expression and activity was paralleled by enhanced p-Akt protein abundance and abrogated by the Akt inhibitor SH-6. Along those lines, transfection with active Akt was followed by increase of SOCE. Most importantly, pharmacological inhibition of either Akt or Orai1 augmented cisplatin induced apoptosis of therapy resistant A2780cis ovary carcinoma cells and virtually abrogated the differences in cisplatin sensitivity between therapy sensitive A2780 and therapy resistant A2780cis ovary carcinoma cells.

The Ca^2+^ channel units Orai1, 2, or 3 [6-9] and their regulators STIM 1 or 2 [12, 13, 15] have been implicated in the resistance to apoptosis, in proliferation, and in migration of tumor cells [16-20, 26-34]. In cervical cancer cells STIM1 silencing abrogates proliferation and induces cell cycle arrest at the S and G2/M phase [32]. SOCE may trigger Ca^2+^ oscillations [35] which regulate a wide variety of cellular functions [36-40] including entering into the S and the M phase of the cell cycle [41, 42] and confering tumor cell survival [43, 44].

In contrast to oscillating cytosolic Ca^2+^ activity, a sustained increase of cytosolic Ca^2+^ activity leads to apoptosis [38, 40, 45-53]. Thus, survival of tumor cells may depend on the delicate machinery underlying oscillating cytosolic Ca^2+^ activity.

Ample evidence has previously been gathered on a role of Akt1 in stimulation of proliferation, inhibition of apoptosis and establishment of therapy resistance [54-62]. Interestingly, Akt1 phosphorylation has previously been shown to be suppressed by SOCE and to be up-regulated by inhibition of Orai1 expression [63]. Thus, the mutual regulation of Akt1 and Orai1 may be part of a negative feedback.

Orai1/STIM1 is known to be up-regulated by the related serum & glucocorticoid inducible kinase [22, 24]. Similar to Akt1, SGK1 is highly expressed in a wide variety of tumor cells [64-67]. SGK1 stimulates cell proliferation and confers cell survival [68-72] and thus actively participates in regulation of tumor growth [65, 73-75]. However, we did not observe significant differences in SGK1 protein abundance between therapy sensitive A2780 and therapy resistant A2780cis ovary carcinoma cells (Suppl. Fig.1). Moreover, the specific SGK1 inhibitor EMD638683 [76] did not significantly affect SOCE in therapy resistant A2780cis ovary carcinoma cells and did not abrogate the differences in SOCE between therapy sensitive A2780 and therapy resistant A2780cis ovary carcinoma cells (Suppl. Fig.2). Thus, under the experimental conditions chosen, Akt1 rather than SGK1 contributes to the therapy resistance of A2780cis ovary carcinoma cells.

The present observations reveal that Akt1 sensitive up-regulation of Orai1 contributes to or even accounts for cisplatin resistance of ovary carcinoma cells. The combined application of cisplatin with Akt1 inhibitors or Orai1 inhibitors may thus overcome therapy resistance of ovary carcinoma. Moreover, the same or similar mechanisms may be operative in other tumor cell types. Different Akt isoforms [77-79], SGK isoforms [65, 80], Orai/STIM isoforms [81-83] or other Ca^2+^ channels [84-89] may confer survival and thus establish therapy resistance of other tumor cell types. The combination of cytostatic therapy or radiation with the respective kinase or channel inhibitors may thus be a novel therapeutic approach to overcome therapy resistance of tumors.

In conclusion, Orai1 is expressed in therapy sensitive A2780 and therapy resistant A2780cis ovary carcinoma cells. Orai1 transcript levels, Orai1 protein abundance and store operated Ca^2+^ entry are all higher in therapy resistant A2780cis than in therapy sensitive A2780 ovary carcinoma cells. The difference in SOCE between therapy resistant and therapy sensitive ovary carcinoma cells is paralleled by and at least partially due to upregulation of Orai1 by Akt. Akt dependent upregulation of SOCE contributes to or even accounts for the therapy resistance.

## METHODS

### Ethics Statement

Investigation has been conducted in accordance with the ethical standards and according to the Declaration of Helsinki and according to national and international guidelines and has been approved by the authors’ institutional review board.

### Cell culture

Experiments were performed in cisplatin-resistant cells (A2780cis) and their therapy sensitive parent cell (A2780) (ECACC catalogue no. 93112519). A2780cis has been generated by exposure to increasing concentrations of cisplatin and is further resistant to melphalan, adriamycin and irradiation [90-92]. The cells were cultured in Dulbecco’s RPMI media, containing 10% fetal calf serum and 1% antibiotic/antimycotic solution.

### Real-time PCR

Total RNA was extracted from ovary carcinoma cells in TriFast (Peqlab, Erlangen, Germany) according to the manufacturer’s instructions. After DNAse digestion reverse transcription of total RNA was performed using Transcriptor High Fidelity cDNA Synthesis Kit (Roche Diagnostics, Penzberg, Germany). Real-time polymerase chain reaction (RT-PCR) of the respective genes were set up in a total volume of 20 μl using 40 ng of cDNA, 500 nM forward and reverse primer and 2x GoTaq® qPCR Master Mix (Promega,Hilden, Germany) according to the manufacturer’s protocol. Cycling conditions were as follows: initial denaturation at 95°C for 2 min, followed by 40 cycles of 95°C for 15 sec, 58°C for 15 sec and 68°C for 20 sec. For amplification the following primers were used (5'->3'orientation):

for Orai1:

fw: CGTATCTAGAATGCATCCGGAGCC

rev: CAGCCACTATGCCTAGGTCGACTAGC

for STIM1:

fw: CCTCGGTACCATCCATGTTGTAGCA

rev: GCGAAAGCTTACGCTAAAATGGTGTCT

for Tbp:

fw: GCCCGAAACGCCGAATAT

rev: CCGTGGTTCGTGGCTCTCT

Specificity of PCR products was confirmed by analysis of a melting curve. Real-time PCR amplifications were performed on a CFX96 Real-Time System (Bio-Rad) and all experiments were done in duplicate. The house-keeping gene Tbp (TATA binding protein) was amplified to standardize the amount of sample RNA. Relative quantification of gene expression was achieved using the ΔCT method as described earlier [93, 94].

### Western blotting

For total protein analysis, cells were harvested with lysis buffer (50 mM Tris, 150 mM NaCl, 1% Triton X-100, 0.5% Na-deoxycholate, 0.4% β-Mercaptoethanol, Proteinase-Inhibitor Cocktail, Roche, Mannheim, Germany). Clarified protein lysate was applied to a polyacrylamide gel and analyzed by western blotting [95, 96]. To this end, 30-50 μg protein of whole cell lysate was incubated with primary antibody for Orai1 (1:1000, Millipore, Bedford, MA, USA, [22]), STIM1 (1:1000, cell signaling, Danvers, MA, USA, [97]), Akt (1:1000, cell signaling, Danvers, MA, USA, [93], Phospho-Akt (Thr308) (1:1000, cell signaling, Danvers, MA, USA, [93]and GAPDH (1:1000, cell signaling, Danvers, MA, USA, [98]). For detection secondary antibody conjugated with horseradish peroxidase (HRP) (1:2000, Cell Signaling, Danvers, MA, USA) was used. Antibody binding was identified with ECL detection reagent (Amersham, Freiburg, Germany). Bands were quantified with Quantity One Software (Biorad, München, Germany [22]). The appropriate band has been defined by using Orai1 overexpressing cells [22, 24].

### Ca^2+^ measurements

Fura-2 fluorescence was utilized to determine intracellular Ca^2+^ concentrations [97]. Cells were loaded with Fura-2/AM (2 μM, Invitrogen, Goettingen, Germany) for 20 min at 37°C. Cells were excited alternatively at 340 nm and 380 nm through an objective (Fluor 40×/1.30 oil) built in an inverted phase-contrast microscope (Axiovert 100, Zeiss, Oberkochen, Germany). Emitted fluorescence intensity was recorded at 505 nm. Data were acquired using specialized computer software (Metafluor, Universal Imaging, Downingtown, USA). Cytosolic Ca^2+^ activity was estimated from the 340 nm/380 nm ratio. SOCE was determined by extracellular Ca^2+^ removal and subsequent Ca^2+^ readdition in the presence of thapsigargin (1 μM, Invitrogen) [99]. For quantification of Ca^2+^ entry, the slope (delta ratio/s) and peak (delta ratio) were calculated following readdition of Ca^2+^.

Experiments were performed with Ringer solution containing (in mM): 125 NaCl, 5 KCl, 1.2 MgSO_4_, 2 CaCl_2_, 2 Na_2_HPO_4_, 32 HEPES, 5 glucose, pH 7.4. To reach nominally Ca^2+^-free conditions, experiments were performed using Ca^2+^-free Ringer solution containing (in mM): 125 NaCl, 5 KCl, 1.2 MgSO_4_, 2 Na_2_HPO_4_, 32 HEPES, 0.5 EGTA, 5 glucose, pH 7.4.

### Determination of apoptosis

To determine apoptosis, 10^5^ cells/100μl in complete DMEM were incubated in 70% ethanol (molecular grade, Sigma) on ice for 30 minutes, centrifuged at 1600 RPM for 3 minutes at 4°C, added to 200μl of hypotonic buffer (0.1% sodium citrate, 0.1% triton X-100, 2mM CaCl_2_, 20U/ml RNAse A in deionized water) together with 24μl/ml Annexin V FITC (Mabtag, Germany) and 50 μg/ml propidium iodide (Mabtag, Germany) as well as incubated on ice in the dark for 60 minutes. The cells were washed once at 1600 RPM for 3 minutes, resuspended in PBS-1% BSA and measured immediately with an excitation wavelength of 488 nm and an emission wavelength of 530 nm (FL-1) versus 585 nm (FL-2) with flow cytometry [100] utilizing a FACS Calibur (BD, Heidelberg, Germany).

### Statistical ananlysis

Data are provided as means ± SEM; *n* represents the number of independent experiments. All data were tested for significance using Student’s unpaired two-tailed *t*-test, one sample *t*-test or ANOVA (Dunnett’s test), where applicable. Results with p<0.05 were considered statistically significant.

## SUPPLEMENTARY FIGURES


