# Supplementary material for: Enhanced Orai1 and STIM1 expression as well as store operated Ca2+ entry in therapy resistant ovary carcinoma cells
Source: Oncotarget. 2014 May 28;5(13):4799–810. doi: 10.18632/oncotarget.2035 (PMC4148100; doi:10.18632/oncotarget.2035)
Supplement: Supplementary file 1 [file oncotarget-05-4799-s001.pdf]

Enhanced Orai1 and STIM1 expression as well as store operated Ca entry in therapy resistant ovary carcinoma cells

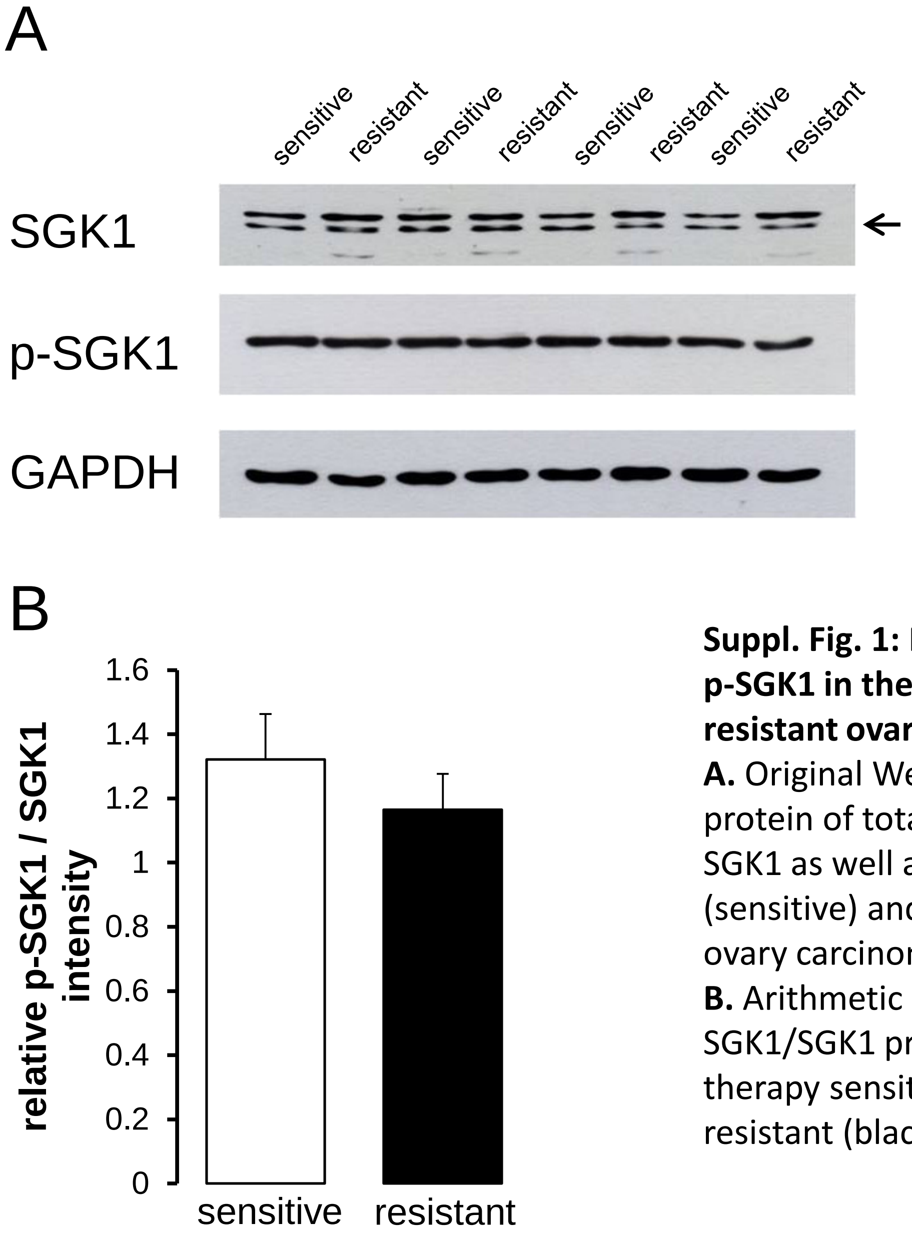

Suppl. Fig. 1: Protein abundance of SGK1 and p-SGK1 in therapy sensitive and therapy resistant ovary carcinoma cells.
A. Original Western blot of whole tissue lysate protein of total SGK1 and phosphorylated p-SGK1 as well as GAPDH in therapy sensitive (sensitive) and therapy resistant (resistant) ovary carcinoma cells.
B. Arithmetic means ± SEM (n = 4) of the p-SGK1/SGK1 protein abundance ratios in therapy sensitive (white bars) and therapy resistant (black bars) ovary carcinoma cells.

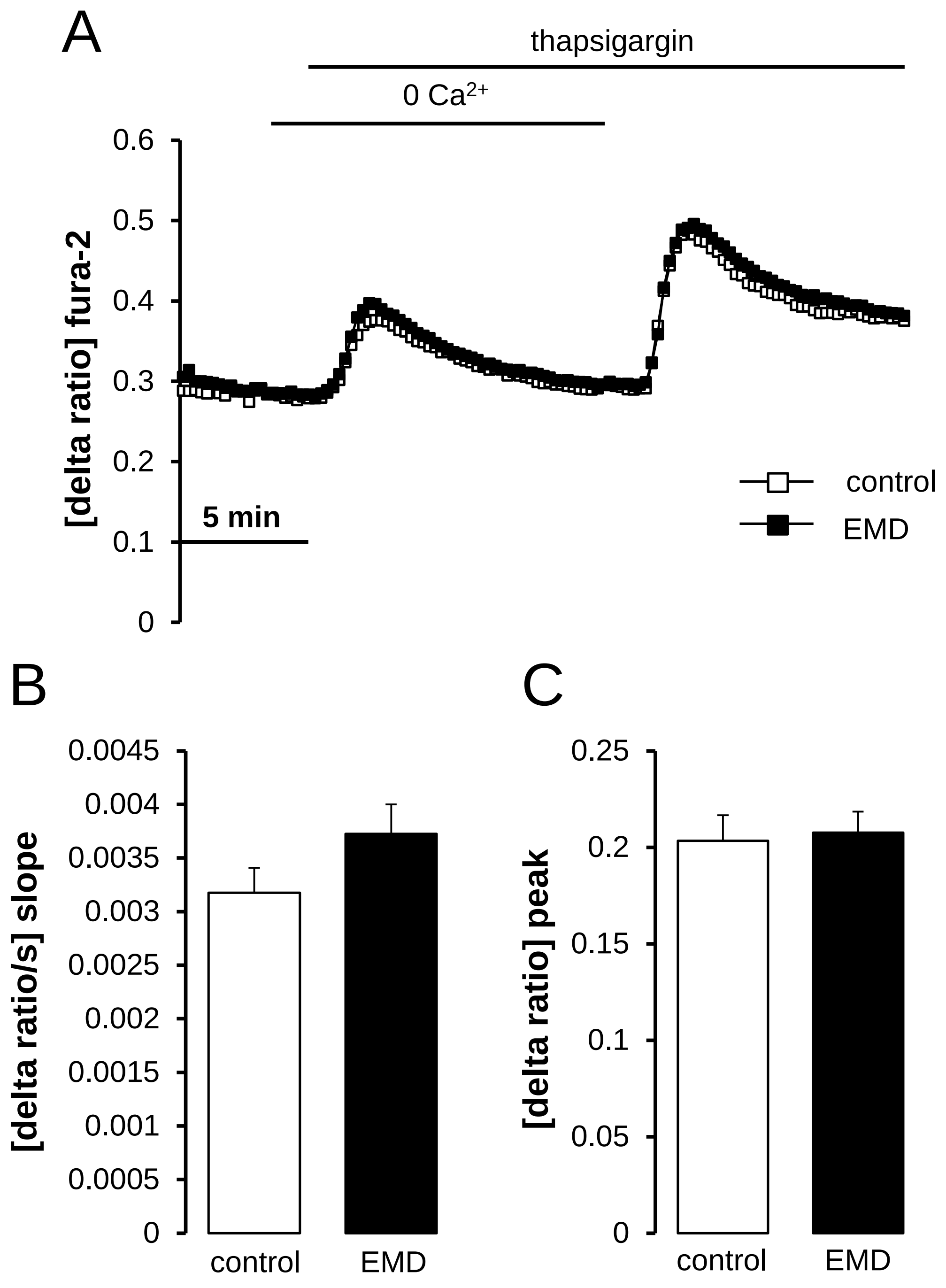

Suppl. Fig. 2: Effect of SGK1 inhibitor EMD 638683 on intracellular Ca^{2+} release and store operated Ca^{2+} entry (SOCE) in therapy resistant ovary carcinoma cells.
A. Representative tracings of fura-2 fluorescence-ratio in fluorescence spectrometry during and after Ca^{2+} depletion with subsequent thapsigargin (1 µM) addition in therapy resistant ovary carcinoma cells without (white squares) and with presence of SGK1 inhibitor EMD 638683 (50 µM, black squares).
B,C. Arithmetic means (± SEM, n = 5, each experiment 10-30 cells) of slope (**B**) and peak (**C**) increase of fura-2-fluorescence-ratio following Ca^{2+} readdition in therapy resistant ovary carcinoma cells in the absence (white bars) and presence of SGK1 inhibitor EMD 638683 (50 µM, black bars).
